# Equity, accessibility, and public health implications of digital platforms delivering real-time air quality information: A technology review

**DOI:** 10.1371/journal.pdig.0001280

**Published:** 2026-04-17

**Authors:** Kayla Schulte, Adam Brighty, William Francis, Andrew Grieve

**Affiliations:** 1 Environmental Research Group, MRC Centre for Health and Environment, Imperial College London, London, United Kingdom; 2 Centre for Environmental Policy, Imperial College London, London, United Kingdom; Tsinghua University, CHINA

## Abstract

According to the World Health Organization, air pollution is the largest environmental risk to global health, with 99% of the world’s population living in areas exceeding recommended guidelines. Providing real-time air quality information through mobile or web-based applications, alongside behavioural guidance, represents a key strategy for reducing individual exposure and improving population health. Such information is delivered at varying geographic and temporal scales and has become increasingly widespread and decentralised. However, there is limited understanding of the quality, characteristics, and potential health impacts of digitally available air quality information and messaging. This study addresses this gap through a systematic technology review of publicly available digital platforms (“channels”) that share real-time local air pollution data. Using the UK as a case study, computational methods were applied to examine how data underpinning existing channels (websites, mobile applications, sensors, etc.) are generated and by whom. Systematic searches of Google, the Google Play Store (Android), and Apple App Store (iOS) were conducted using SerpAPI and predefined search terms. In total, 146 channels met inclusion criteria and were analysed. Channel metadata were used to identify trends in channel types, evolution over time, and emerging patterns across the digital information landscape. The review also compares differences in available information across channels but is limited to those accessible in the UK and excludes real-time social media data due to cost and access constraints. Finally, the study considers demographic and social factors influencing access to air quality information and its exposure-reducing benefits. These findings contribute to understanding the production and use of digital air quality information with global public health relevance.

## Author summary

Air pollution is the world’s biggest environmental threat to health. The World Health Organization estimates that 99% of people globally live in places where air quality exceeds safe levels. Sharing real-time air pollution information, alongside advice on how to reduce exposure, can help protect human health. This information is now widely available through websites, mobile apps, and other digital tools. However, because these tools are created by many different organisations and use varied data sources, it is unclear how consistent, reliable, or useful they are for protecting public health. We aimed to better understand the digital air quality information available to the public. Using the UK as an example, we systematically reviewed publicly available digital platforms, known as “channels,” that provide real-time local air pollution information. Using automated searches, we identified 146 channels that are freely available to the public. We found clear differences in how air pollution data and health messaging are presented, and we discuss implications around who is most and least likely to benefit from this information.

## 1. Introduction

Air pollution is the leading contributor to global disease burden, accounting for over 8 million deaths in 2024 [1,2]. The increasing availability of internet connected technologies (ICTs), including mobile phones, sensors and computers promises to expand the reach of health protective information at orders of magnitude greater than has yet to be observed [3,4]. Mounting evidence from digital health studies, including those implementing randomised control trials (RCTs), demonstrates that the delivery of information about air quality conditions, or ‘AQ information’, coupled with practical behavioural advice can lower exposures and improve health outcomes at the population level, with examples ranging from reduced asthma attacks to less emergency room visits associated with adverse respiratory and cardiovascular incidents [5,6,7,8].

The term air quality (AQ) channel is used as a comprehensive label for web or app-based platforms that deliver air pollution information in near real-time, representing a convergence of three categories of innovations: advances in air pollution data generation, air pollution modelling techniques, and the advancement of ICTs that can promote digital health literacy [9,10]. In addition to AQ specific channels, digital health interventions such as mobile apps aimed at individuals with asthma or other chronic health conditions are integrating real-time air pollution data and behavioural messaging, with the number of these apps more than doubling between 2011 and 2013 [11,12]). A lack of consistency surrounding data quality and health messaging presents barriers surrounding how to interpret air quality information [13,14]. Such discrepancies increase the likelihood of inaccurate information and can contribute to confusion or unintended outcomes amongst users. With academics and practitioners calling for greater standardization or the implementation of a universal air quality index (AQI) [9], this motivates the need to understand the risks and benefits associated with ‘freely available’ AQ information, referring to information that is that is not behind a paywall however, requires ownership or access to an internet-connected device.. This can be achieved by conducting a systematic technology review to synthesize the contemporary landscape of freely available AQ information.

Despite the existence of publicly available real-time air pollution data and health protective advice, evidence suggests that this information is not widely taken up throughout the UK population [15]. Concurrently, both access to and use of digital health technologies has been found to be higher for individuals who are White, English speaking and have no disabilities [16]. Cochrane’s PROGRESS PLUS serves as a helpful framework for understanding how different sociodemographic dimensions with equity-related implications, including geography, ethnicity, age, gender and sex, education, socioeconomics and sociocultural factors, can affect patterns of interaction with digitally delivered, health-protective air pollution information [17]. The analytical relevance of this framework is reinforced by recent evidence of sociodemographic characteristics, specifically having higher education qualifications or caring for someone with a health condition, being linked with greater odds of adopting behaviours in response to digital air quality information [15].

Acknowledging the persistence of digital and information inequalities, this review responds to the need to consider how the current AQ information landscape may exacerbate disparities, as certain population groups are more likely to access and respond to digitally delivered health-protective information [15,18]. Recent evidence points to the causal effects of both digital access and usage on healthy aging outcomes in China, with impacts of digital divides observed across physical, cognitive, emotional and social outcomes [19]. Further, sources and dominant pollutant types vary from country to country, as well as seasonally and geographically [20]. The most recent UK National Atmospheric Emissions Inventory (NAEI) cites PM_2.5_, PM_10_ and NO_2_ as major pollutants of concern for public health in the UK, with domestic and industrial combustion, construction and demolition and road transport as dominant sources [21]. Addressing these complexities necessitates a sensitive methodological approach that integrates computational, statistical, and content analysis to support a comprehensive exploration of AQ channels’ operation and impact across the UK.

This review aims to identify characteristics of AQ channels available to individuals across the UK, in an effort help assess the equity and inclusivity of AQ information distribution for health protective aims. It adopts the UK as a case example to evaluate how AQ data and information underpinning existing AQ digital channels is generated, by which entities, and how it used. As such, this review can supply timely information around AQ channels’ role in shaping environmental awareness and public health outcomes across varied socio-economic and demographic contexts.

## 2 Methods

This review was completed in line with PRISMA guidelines and did not require registration with the PROSPERO protocol as no direct health impacts were reported. Ethical approval was not required.

### 2.1 Study design

This project uses both systematic review and metadata analysis approaches to gather data about the UK AQ information systems landscape, advancing methods used by Schulte [22]. Based on a traditional systematic review format, this study also incorporates features from literature reviews and HTML extraction-based studies, which are explained in sections 3.2 – 3.6.

Adaptation of this traditional review format was necessary because of the new forms of digital AQ data and metadata available across modern-day AQ information delivery platforms, moving from predominantly web browser-based channels to include digital phone and tablet applications. These include app download data, Domain Name System (DNS) server records^3^, and HTML data analysis. These data can help contextualise and characterise ‘transactional’ engagements with the digital AQ information landscape, but care must be taken to not interpret them superficially. User analytics data are produced through complex sociotechnical systems and acknowledging helps ensure results from this review are not extrapolated out of context [23,24]. Cochrane’s PROGRESS-PLUS framework is also leveraged to identify and interpret equity-relevant data resulting from this review.

The search engine query software SerpAPI was employed to investigate digital AQ channels across Google web searches, Google Play Store (Android), and Apple App Store (iOS). This allowed for a consistent, fast, and thorough search of channels across all digital delivery platforms. X (formerly Twitter) API was also used for X searches.

### 2.2 Data sources

Google web search was chosen as the internet search engine for the SerpAPI investigations, as it holds 90% of the UK search engine market share [25,26]. Similarly, the investigation of air quality applications across desktop and mobile devices focussed on the iOS and Android platforms, as they retain 99% of the UK smartphone market [25].

The X API did not allow for the searching of user accounts according to specific search terms. Therefore, this study employed a manual strategy using X’s advanced search function, retrieving the top 20 accounts associated with the search terms. Unique accounts were identified and investigated manually to determine whether they met the study’s inclusion criteria.

Social media platforms including Youtube, TikTok and Meta products (Facebook, Instagram) presented greater difficulties in garnering information, and as a result were not included [27]. Both radio and TV channels are also important within the UK broadcast landscape. Data mining across these platforms, however, was not possible due to the lack of searchable archives. Outdoor digital displays and billboards were also excluded, as investigating these require approaches beyond the scope of this investigation.

### 2.3 Search strategy

The search terms used in the study to generate the list of publicly available, UK-based AQ channels were adopted from Schulte [22] (see Appendix 1.2). The authors completed a screening studied to determine whether the geographic location (IP address) impacted the search results; city-specific results were found. Based on this, the SerpAPI searches were replicated across both city, county, and shire levels. This also ensures the searches were not included for only urban regions of the UK.

The UK cities list was obtained from UK Government official webpages (UK Cabinet Office, 2022), but a wide range of sources were needed to define UK counties. Historical counties were also excluded from the list:

- In England, only current ceremonial counties were included.
- In Scotland, registration counties were used instead of Lieutenancies.
- In Northern Ireland, all counties were included.
- In Wales, 22 counties and county boroughs were used.

SerpAPI could not retrieve data in 49 locations as their location names were not included within SerpAPI’s ‘supported location list’. To combat this, postcodes to match the county town or the London borough were used. Four minor Scottish counties were not found within the supported location list using either county names or postcodes. 207 locations were included; a complete list of locations and search terms are included in S1 Table and S1 Text.

For SerpAPI searches across the Google search engine, results were limited to 100 returns. Each Google search page returns 8 – 10 results (Google documentation, 2023). The same search terms were queried across the Google Play and Apple App stores, although it was not possible to set the geographic location for these platforms. Reproducible scripts are available via GitHub at github.com/arg02/uk-digital-landscape/, while all search parameters can be found in S1 Text and S2 Text.

### 2.4 Selection criteria for AQ channels

The criteria for filtering which AQ channels to include in the review are summarised in Panel 1:
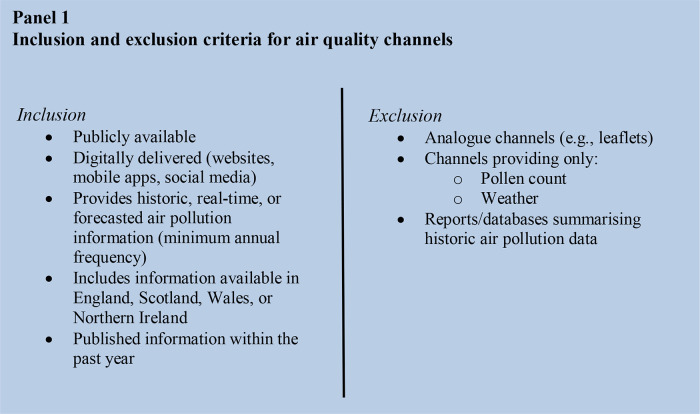


A three-step process for refining search results was implemented. The first step used SerpAPI to remove duplicate results from the dataset. The second step used a ‘crosswalk’ to cross-reference each AQ channel’s description and extracted HTML text against a dataset of 50 common terms found across known AQ channels. A score was generated for the number of terms included in each unique search result. This was used as a screening exercise to indicate which channels were most likely to meet the AQ channel screening criteria. Complete details of this process are described in Appendix 2.1. The final step relied on manual checks to review each entry in the dataset to confirm that an AQ channel met the inclusion criteria in Panel 1. The retained entries were then populated with metadata.

### 2.5 Metadata categories

Metadata were collected for each AQ channel retained through the initial screening process. A complete list of the metadata fields can be found in Appendix 3.1. A proportion of the metadata fields were filled through the SerpAPI HTML extraction process. Other fields were populated by matching key terms with the either the app’s description or HTML text on the website homepage. Remaining metadata fields were filled via manual searches. Calculating the AQ channel launch date for websites was predominantly based upon domain registration dates, obtained using whois.com. In some cases, the launch date was listed on the website or based on the earliest data shared through the website. App launch dates were returned automatically by SerpAPI.

Categories for reporting how air pollution was presented across each channel was informed by the two dominant methods for AQ measurement: modelling and equipment-based measurement [28]. Modelling combines multiple datasets (e.g., emissions inventories, weather, traffic patterns) to estimate pollution concentrations at different spatial and temporal resolutions. Measurements are derived from government monitoring stations, diffusion tubes, or lower-cost sensors. In the UK, 150 automated monitors collect hourly data on pollutants like PM_10_, PM_2.5_, SO_2_, CO, NO, NO_2_, and O_3_, supported by quality assurance protocols [29]. Metadata obtained for each AQ channel indicates the mode through which air pollution levels were estimated.

### 2.6 Framework for analysis

The resulting dataset formed the basis of the content used to evaluate the UK-wide AQ channel landscape and support the characterisation of channels available to the domestic population. Data collection and management, as well as the generation of summary statistics and graphs, were performed using RStudio, SerpAPI and X (formerly Twitter) API. R Studio packages used include: tidyverse, jsonlite, readxl, xlsx, httr2, httr, lubridate, rvest, data.table, urltools, rlist, r.utils, RSelenium, and XML.

### 2.7 Role of the funding source

The funder of the study had no role in study design, data collection, data analysis, data interpretation, or writing of the report.

## 3. Results

**Fig 1:**
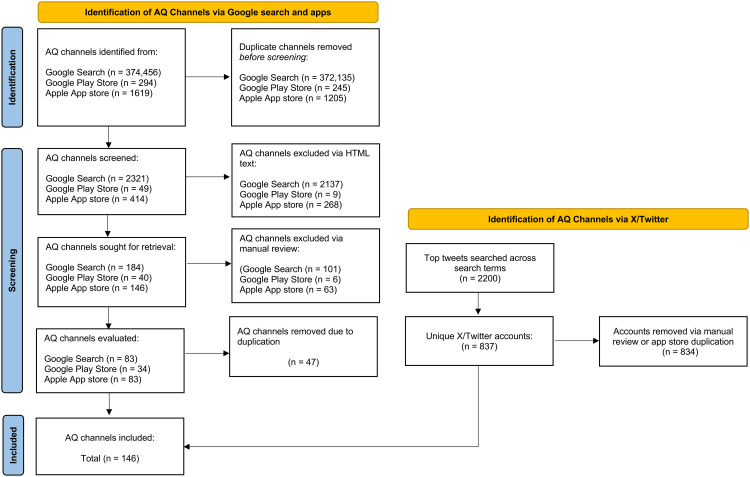
Refinement of systematic technology search results.

376,369 items were returned by the initial search of Google, the Google Play Store, and the Apple App store, from which 2784 AQ channels were initially identified. 837 X/Twitter accounts were identified from the tweet search, from which 3 AQ channels were identified. After screening by the inclusion criteria, 146 AQ channels were selected (Fig 1). Certain AQ channels were multi-platform, with 83 (56.8%) available through websites, 83 (56.8%) available as Apple apps, and 34 (23.3%) available as Android apps. Metadata was collected for all 146 AQ channels, however, download count was only available for the 22 AQ channels delivered via the Google Play Store. AQ channel launch date ranged from 2001 to 2023, however most channels were registered from 2017 onwards (53.4%).

### 3.1 AQ channel data providers and data producers.

**Fig 2:**
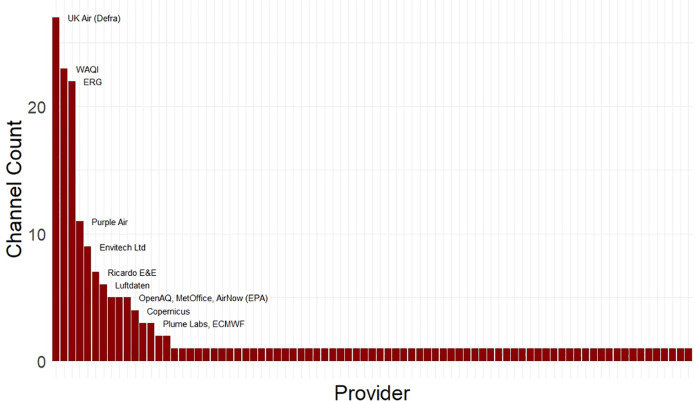
Largest AQ data providers in the UK.

**Fig 3:**
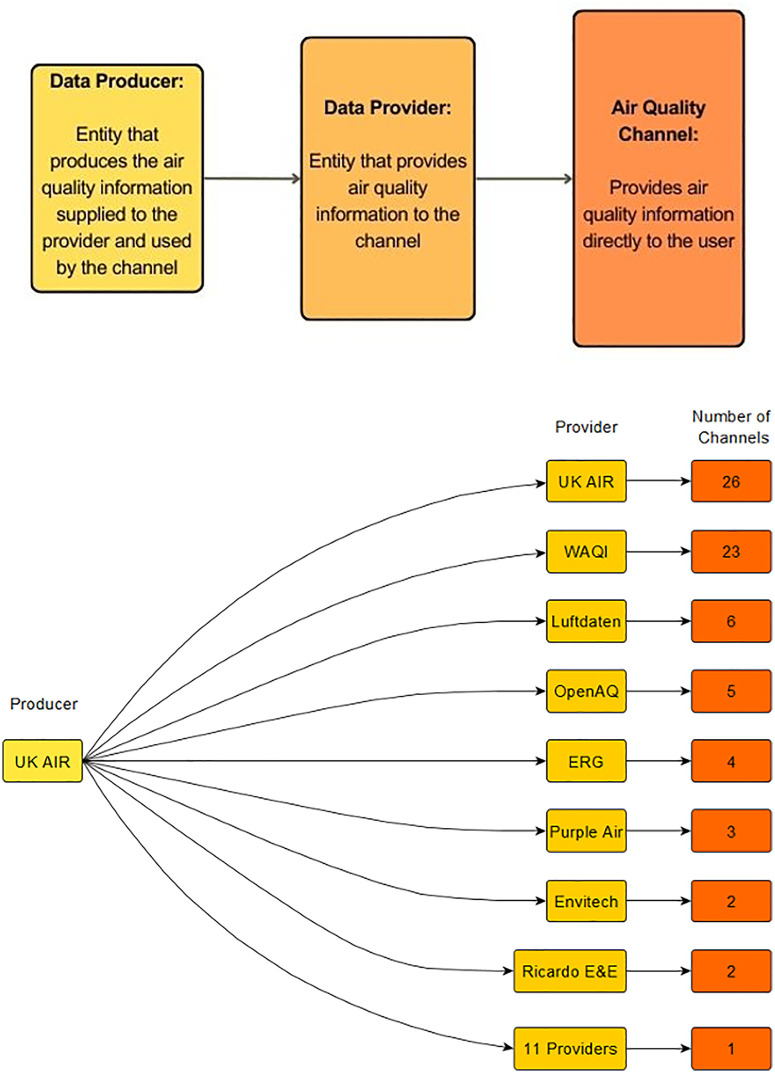
Data pipeline from UK AIR, a data producer, to a range of data providers and channels. Data pipelines from other providers and tabulated versions are found in the Appendix (4.1 and 4.2).

Many of the channels sourced data from a common data provider (58.2%) or multiple data providers (46.6%), with multiple providers often observed from channels delivering a forecast alongside data from sensors or regulatory monitors (33.8%). The data provider most frequently used by channels was the Department for Environmental Food and Rural Affair’s (Defra) air information resource, UK Air, followed by the World Air Quality Index (WAQI) and Imperial College London Environmental Research Group (Imperial ERG) (Fig 2). The data providers do not necessarily produce the data used by channels, as indicated in the data pipeline shown in Fig 3. For example, the WAQI represents a collation of data measured by multiple producers and does not produce any data itself. In contrast, both UK Air and Imperial ERG themselves produce the data they provide to channels, and UK Air also functions directly as an AQ channel. There were also 22 channels that did not clarify the providers or producers of the air quality data underpinning the information they delivered.

### 3.2 AQ channel information provision

The most common format of AQ information presented across the channels reviewed was an AQ index (AQI) (80.1%), followed by nitrogen dioxide (NO_2_) (75.3%), 2.5µm particulate matter (PM_2.5_) (74.7%) and 10µm particulate matter (PM_10_) (71.2%). When an AQI was provided, it was most often the U.S. AQI established by the U.S. Environmental Protection Agency (43.6%), or the Daily AQI established by Defra (41.9%). Certain channels included reference to the 2021 World Health Organization (WHO) exposure guidelines. Health guidance accompanying AQ information was limited, with most AQ channels neither outlining exposure-reducing behaviours nor identifying sensitive groups (58.9%). AQ levels or readings were primarily delivered for a specific point (70.5%), with a minority of channels reporting AQ levels across a larger 10x10km resolution (8.2%) or at other geographical resolutions. Most channels updated their AQ information hourly (69.9%).

Metadata indicating patterns of user access and engagement was available for a subset of the AQ channels. App downloads were obtained when available via the Google Play store as a proxy for evaluating AQ channel reach or popularity. These data are presented alongside additional metadata to assist with comparing results from the subsample with the complete AQ channel dataset (Table 1).

**Table 1:** Reach dataset summary statistics.

	Complete AQ Channel Dataset	Subsample
Number of AQ Channels	146	22
Percentage of total dataset	100%	15%
Channel launch date range	2008-12-22 to 2022-08-14	2001-05-09 to 2023-09-20
Available through website platform	88%	55%
Available through phone	63%	100%
Available through tablet	50%	82%
Available through television	1%	5%
Available through smartwatch	14%	32%
Available through Android platform	27%	100%
Available through Apple IoS platform	57%	68%
Offering as an API	16%	14%
Using a model data source	34%	27%
Using a monitor data source	64%	59%
Using a sensor data source	19%	32%
Provides a forecast	34%	36%
Provides historic data	51%	32%
Provides PM_10_ data	71%	82%
Provides PM_2.5_ data	75%	77%
Provides PM_1.0_ data	7%	5%
Provides nitrogen dioxide data	75%	68%
Provides sulphur dioxide data	44%	27%
Provides ozone data	51%	41%
Provides carbon monoxide data	32%	18%
Provides nitric oxide data	16%	9%
Provides nitrogen oxides data	11%	0%
Provides black carbon data	3%	0%
Provides benzene data	3%	0%
Provides allergen data	7%	5%
Provides exposure reducing behaviour messaging	40%	68%
Provides sensitive group identification	36%	68%
Provides a paid option	15%	18%

## 4. Discussion

This systematic technology review identified freely available AQ channels providing information about air pollution and health effects, comprising the UK digital AQ information landscape. A total of 146 channels were recorded across three platforms and were analyzed to generate insight around common and divergent characteristics pertaining to channel infrastructures, in addition to characteristics of the information provided.

### 4.1 Mapping the expanding AQ info landscape

**Fig 4:**
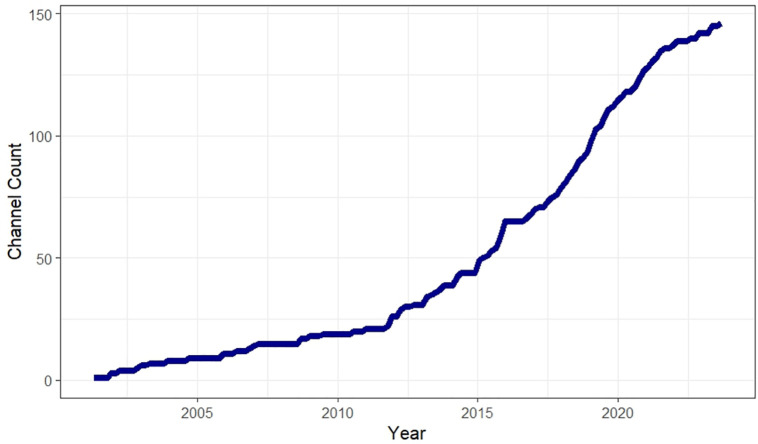
Cumulative AQ channels launches over time in the UK.

The number of currently active channels grew from 2 to 146 between 2001 and 2023, alongside an increase in the number and types of active organizations producing AQ data. Notably, this includes a shift away from the government serving as the sole provider of AQ information, and the introduction of commercial companies into the AQ digital landscape. Spikes in the number of channels entering the landscape were observed in 2001, 2012, 2015, 2019 and 2021, while 53.4% of channels are thought to have been generated in 2017 or later (Fig 4). A potential driver of the spikes includes events such as the ‘dieselgate’ scandal in 2015, which may have contributed to greater public interest in AQ issues [30]. In addition, the growth of the smartphone and mobile application market in the UK over the past 15 years is likely linked to the growth of the AQ channel landscape [11]. This review confirmed an increase in mobile app-based channels compared to those delivered via traditional websites. The greater number of Apple IoS apps is likely related to the ease of developing iOS apps through a developer-friendly interface.

The opportunity to generate revenue through advertisements and paid services is hypothesised to have driven the proliferation of free apps. This aligns with commercialisation trends throughout the AQ information sphere, as informational tools including AQ channels and sensors, as well as personal exposure reducing technologies such as masks and air filters are marketed to help individuals avoid or reduce exposure to pollution [31,32]. Considering evidence of a causal link between both access and usage of digital technologies and less healthy aging trajectories [19], further research is warranted to determine how such tools and technologies may sustain or exacerbate existing exposure inequalities, as those who can afford or maintain capacities for using these products may experience different level of protection from pollution compared to those who do not.

To examine the reach of AQ channels, a subsample was generated and included 22 of the channels available as mobile applications that maintained download-related metadata (see Table 1). The subsample highlighted that channels which included the topic tag ‘weather’ in the app store were the most frequently downloaded, where weather-focussed apps were downloaded 100 million times compared with 6 million times for AQ-specific apps downloaded. For AQ-specific apps 5 million of the downloads were associated with the AQ channel *IQAir*. It is plausible that the advertising budgets of commercial channels contribute to greater number of downloads, when compared with government or independent AQ channels. Further, the finding that weather focused channels are more frequently downloaded suggests that individuals may arrive at AQ information via alternative entry points or topics of interest. Findings from the subsample highlight both the existing and potential reach of AQ information across the digital landscape, potentially exceeding 100 million people. However, they also reveal potential disparities in exposure reductions for certain groups who are less likely to engage with these channels, which is addressed in greater detail in section 5.4.

### 4.2 Technological infrastructure and mechanisms underpinning the UK digital AQ landscape

Fig 4 showed a schematic of the production, provision and display of AQ data across AQ channels. The results highlighted that the data underpinning the content displayed via a particular channel’s user interface are often from a third-party *data provider*. Further, the results also indicated a selection of common third-party data providers supply data for the majority of AQ channels. Another layer of complexity is introduced as the results demonstrated that data providers are not necessarily *data producers*. Data producers supply data they have been involved in generating and make it available through their own AQ channel interface or by way of download or increasingly through an API. Reviewing what types of AQ data channels present (i.e., measured or modelled data) helps to clarify why the AQ data pipeline is structured in this manner. Most channels present measured data from regulatory monitors, followed by sensor-based sources. This is presumably a function of open access regulatory monitoring data following the governments, and other publicly funded data producers including universities’, commitments to the open data ethos.

A ‘nesting doll’ effect can be observed across the AQ information landscape, where looking beneath a channel’s unique user interface layer, and past the data provider layer, reveals common core of data producers underpinning the informational content being presented by dozens of different channels. The top five data producers are all government funded operations. Tracing the assimilation of publicly funded, multi-institutionally generated data like Defra’s AURN monitor data, or the London Atmospheric Emissions Inventory (LAEI) and European CAMS or SILAM model outputs, into AQ channels is further complicated by proprietary restrictions, ‘black boxing’, and missing data source attributions. 22 channels did not indicate which data providers or producers underpin the information they share. In addition, channels presenting modelled air pollution estimates syphon data through providers or directly from data producers, before undergoing proprietary adjustments and incorporation into black-boxed, modelled estimates then presented monolithically. The results also signal a growing trend of channels offering paid monthly or annual subscriptions for accessing data outputs. The ‘repackaging’ and sale of data products using publicly funded environmental datasets raises ethical questions around the expropriation and used of taxpayer-funded public informational goods to generate commercial profits. In today’s information economy, more explicit stipulations around the use or inclusion of publicly funded and produced AQ data may be necessary to preserve the accessibility and longevity of this public resource.

### 4.3 Air pollution data formats and messaging

The format of the information and messaging content delivered across the resulting AQ channels demonstrated several key trends. Most channels delivered AQ information at hourly intervals and a specific latitude and longitude point location. In addition, certain channels reported pollution levels at multiple geographic resolutions when they derive data from both sensors or monitors and models. This highlights the possibility where an individual has ‘location services’ activated and an assumption is made that the reported AQ reading corresponds to one’s precise location, when it rather pertains to data from the closest monitor, sensor or from a lower resolution model (up to 10km^2^).

Metadata from the channels reinforced a trend towards more spatially and temporally granular data delivery, with certain channels reporting data in near real-time (i.e., 1–5-minute averages). Most channels appear to either mirror or directly copy the UK government’s daily air quality index (DAQI) format for delivering AQ readings and associated health messaging. DAQI or AQI formats were maintained across channels that reported at 1–5-minute intervals, despite an incongruence with hourly AQI averaging periods.

Many of the channels examined through this review were generated outside the UK, and in instances where the channel was not automatically configured to adapt content to match the UK DAQI based on geolocation data, they most often presented data according to government agency formats such as the U.S. AQI or European CAQI. Non-governmental, channel-specific AQI indices were also present in the results, contributing to the heterogeneity of the AQ information landscape. AQIs are designed for short term alerting, while WHO guidelines reflect medical evidence reinforcing health damages associated with pollution exposure, even at lower levels, over the longer term. Discussions surrounding potential confusion arising due to variations in AQI from country to country introduce the prospect of a universal AQI. Despite the inclusion of WHO guidelines across certain AQ channels, results from this study suggest the global adoption of a universal AQI is unlikely, particularly as AQ information delivery patterns are highly geographically and institutionally disaggregated.

Further, disparities in pollution concentrations between cities around the world is significant. A universal AQI would dilute the risk messaging for regions like the UK where pollution concentrations are comparatively low [1]. Diluting air pollution and health risk messaging by way of a universal AQI could have far reaching implications, ranging from population health burdens to diminished pressure for stricter air pollution regulations throughout the global north. A universalist approach to information delivery also runs counter to findings from the AQ communications literature, which reinforce the improved efficacy and uptake of information that is more personalized [13]. If a universal AQI were developed however, it should include a long-term exposure dimension to reinforce the cumulative health risks of exposure at lower concentrations.

This analysis demonstrates that drivers of variation observed across AQ channels warrant greater clarity and transparency. Temporal and spatial specificities of data sources or averaging applied by channel providers introduce the greatest risk for misinterpreting the relevance of air pollution concentrations presented to an individual in time and space. Frameworks or global requirements for reporting data sources, crucially spatial and temporal dimensions, are essential for interpreting information conveyed by AQ channels. Importantly, 14% of the channels did not provide any form of guidance or data interpretation. Failure to include context alongside reported air pollution levels misses a fundamental opportunity to render otherwise arbitrary information meaningful and actionable for different audiences [13,14].

### 4.4 Equity implications

Using Cochrane’s PROGRESS PLUS framework, our findings highlight how existing infrastructural features of AQ channels may reinforce inequities in access, understanding, and use of exposure-reducing information. The results found that a majority of AQ channels (68.5%) presented pollution levels for a specific geographic point. This relies on the user’s ability to interpret the relevance of a reading for their geographic location. For example, AQ channels commonly present readings from the ‘nearest’ sensors or monitors, which can be over a mile from the user’s location, particularly if they reside in more remote areas. While pollution levels are generally higher in urban settings, AQ channels broadly lack clarity regarding the geographic resolution of the pollution readings they present, which can contribute to place-related disparities in actionable information. Further, most channels (69.8%) provided information at hourly intervals. It is possible for hourly reading to meet national or even WHO guidelines, while breaching annual averages that associated with poor health outcomes over the longer term. These channel features rely on users interpretive ability and understanding of short vs. long term exposure impacts, which can require higher levels of education or scientific literacy, potentially disadvantaging users with fewer educational resources or limited familiarity with scientific principles.

Equity concerns are further amplified by limited health and behavioural guidance accompanying AQ information. Most channels do not clearly identify exposure-reducing actions or specify who may be more vulnerable to pollution (40 and 36% respectively). Where “sensitive groups” are referenced, definitions are often fixed and top-down, leaving little room for individuals to recognise their own circumstances or intersecting vulnerabilities [14]. This lack of personalisation may particularly exclude people whose risk profiles fall outside standard categories, such as those with multiple conditions, caring responsibilities or those associated with other PLUS factors [17]. Lastly, there is mounting evidence reinforcing how individuals with high technological literacy are more likely to experience benefits from digital health platforms [19]. Younger or wealthier individuals who are more likely to have higher access to AQ channels receive more marketing of pollution mitigation strategies such as masks or air filters, which can have a direct effect on exposure and ultimately health outcomes [33,32]. Together, these features suggest that current AQ information systems may better serve already advantaged populations, underscoring the need for more inclusive, interpretable, and adaptable digital AQ communication to avoid widening existing inequalities.

### 4.5 Limitations and future research

As certain AQ channels were mobile apps, the search methodology could not be fully automated. Combined, Apple’s App store and Google’s Play Stores account for > 90% of app downloads globally [10]. However, Apple does not provide usage information whilst Google provides only install ranges for each app. Thus, the researchers had to manually collect data and validate channel characteristics. This involved downloading app-based channels, which was time consuming and not necessarily replicable for larger datasets.

The researchers did consider third party services such as Sensor Tower and SimilarWeb which provide app usage metrics. However, such services rely on indirect signals such as app store rankings, social media sentiment analysis and advertising impression data to build proprietary inference models. To ensure transparency, and also because these types of inference models are particularly uncertain at low download volumes (100s-1000s) the researchers decided not to use these types of services for this analysis [33,34].

Precise launch dates for most AQ channels were unavailable, and DNS registration dates were used as a proxy. It is unknown whether the DNS registration dates correspond directly with channel launch date. The focus on dissemination of AQ information in digital spaces overlooks digital inequality, as certain groups including older individuals, non-native English speakers, are less likely to engage with digital information sources [15]. The AQ information landscape warrants further research regarding the interplay of digital and non-digital information sources for engaging diverse groups. Future research should explore social and broadcast media, including TV and radio, and extend AQ channel analysis beyond the UK.

Evaluating real-time AQ information on social media was beyond the scope of this study due to cost and access constraints. Nevertheless, research highlights social media as a key information-sharing platform in the UK, with 63% of the population using it daily and 47% relying on it for news [35]. Influencers on Twitter/X, Instagram, TikTok, and YouTube present potential avenues for AQ information dissemination, as do closed messaging platforms like WhatsApp, which played a key role in COVID-related communication [36,37]. While ICTs have expanded the AQ information landscape, the growth of social media exponentiates the volume of individuals that could be, and already are, interacting with health protective air quality information.

As user interactions are often algorithm-driven, distinguishing between direct and mediated communication is crucial. Questions surrounding trust and quality of information sources are likely to impact whether individuals interact with AQ information, and whether the information is more harmful than helpful. Despite these limitations, social media presents an unprecedented opportunity to evaluate social and behavioural outcomes associated with large scale air quality health messaging campaigns. Further research is warranted in this domain, particularly by employing social network analysis approaches and methods for analyzing visual media.

## 5 Conclusion

This systematic technology review maps the evolving landscape of freely available digital AQ information channels in the UK, highlighting growth in both channel numbers and provider diversity. The expansion beyond government-led initiatives to include commercial entities reflects shifts in the dissemination, commercialization, and modes for accessing such information. The increasing role of mobile apps, particularly those integrated with weather-related content, underscores the importance of understanding how and why individuals engage with AQ information, often via alternative entry points of interest like wellbeing or athletics.

Analysis of technological infrastructures reveals complexities in data generation and sourcing, with a few publicly funded producers supporting numerous AQ channels. Open access to government AQ data has expanded public reach, yet transparency, proprietary modifications, and commercialisation raise ethical concerns. Inconsistencies in AQ data presentation, such as spatial and temporal discrepancies and AQ index variations, highlight the need for clearer frameworks to enhance interpretability and relevance.

Future research should explore social media and broadcast platforms’ roles in AQ information dissemination, given their influence on social norms and health behaviours as well as their expansive reach. Simultaneously, addressing digital inequalities in AQ access is crucial to ensuring vulnerable populations are not disproportionately excluded from digital health protections. This methodology has proven effective at identifying characteristics of the AQ info landscape in the UK and establishes a framework to be applied to other national or international contexts. Future applications can support knowledge building surrounding whether these emergent digital health platforms are benefiting global populations disproportionately, alongside evaluation of cultural contexts that may affect AQ channel diffusion patterns. As air pollution remains a major public health issue, enhancing the transparency and accessibility of digital AQ information could lead to significant advancements in informed decision-making and equitable health outcomes at both individual and population levels.

## Supporting information

S1 Checklist PRISMA checklist. From: Page MJ, McKenzie JE, Bossuyt PM, Boutron I, Hoffmann TC, Mulrow CD, et al. The PRISMA 2020 statement: an updated guideline for reporting systematic reviews. BMJ 2021;372:n71. https://doi.org/10.1136/bmj.n71. This work is licensed under CC BY 4.0. To view a copy of this license, visit https://creativecommons.org/licenses/by/4.0/.
(DOCX)

S1 Fig Refinement process & common AQ channel descriptors. (DOCX)

S1 Table Location list. (DOCX)

S1 Text Search terms. (DOCX)

S2 Fig Provider-specific AQ data pipelines. (DOCX)

S2 Table Tabulated AQ data pipelines. (DOCX)

S2 Text Metadata list. (DOCX)
