# Supplementary material for: Equity, accessibility, and public health implications of digital platforms delivering real-time air quality information: A technology review
Source: PLOS Digit Health. 2026 Apr 17;5(4):e0001280. doi: 10.1371/journal.pdig.0001280 (PMC13089882; doi:10.1371/journal.pdig.0001280)
Supplement: S1 Fig — (DOCX) [file pdig.0001280.s002.docx]

# S1 Fig. Refinement process & common AQ channel descriptors


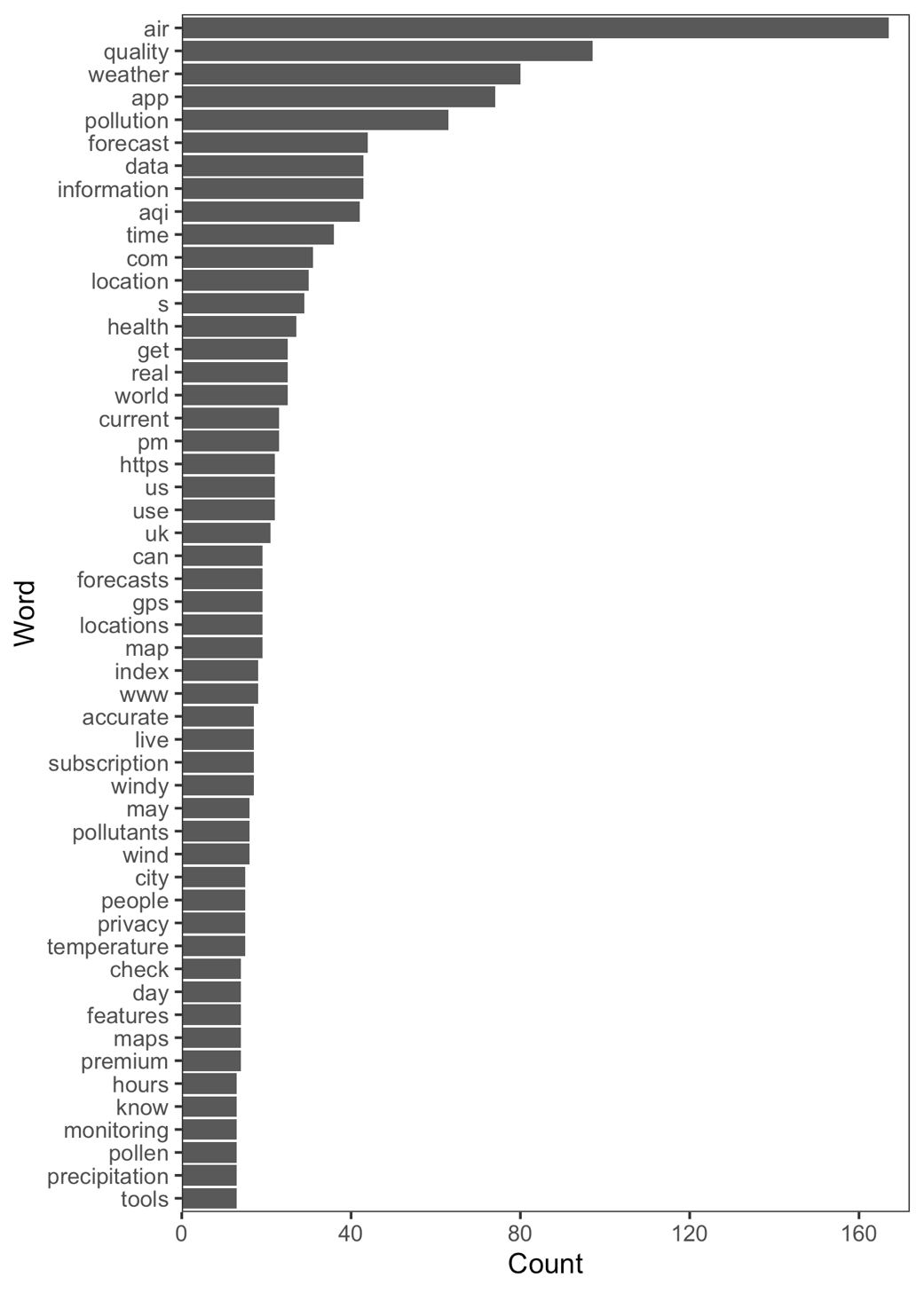


The most commonly featured terms across known AQ channels were used to help refine the dataset resulting from the systematic search (see Fig 4). The 52 search terms appearing most frequently in air quality related apps on the google playstore were loaded and for the html text corresponding to each unique url, and each unique domain, a 1 or 0 was recorded, indicating whether the html text did or did not contain each search term irrespective of case. A column was then added indicating the sum across all search terms for each unique url and unique domain.

To filter out duplicate domains (e.g. <https://www.breathelondon.org> and <https://breathelondon.org/>) domain which matched after the removal of the ‘https://’ prefix, and the ‘www.’ were identified and only one copy of the domain retained. Domains for which the suffix differed (e.g. https://www.air-quality.com and <https://www.air-quality.org.uk>) were retained as these reflect different websites.
