# Supplementary material for: Equity, accessibility, and public health implications of digital platforms delivering real-time air quality information: A technology review
Source: PLOS Digit Health. 2026 Apr 17;5(4):e0001280. doi: 10.1371/journal.pdig.0001280 (PMC13089882; doi:10.1371/journal.pdig.0001280)
Supplement: S1 Table — (DOCX) [file pdig.0001280.s003.docx]

# S1 Table. Location list

| **Location** | **County/Largest Town** | **Postcode** |
| --- | --- | --- |
| Bath,England,United+Kingdom |  |  |
| Birmingham,England,United+Kingdom |  |  |
| Bradford,England,United+Kingdom |  |  |
| Brighton+&+Hove,England,United+Kingdom |  |  |
| Bristol,England,United+Kingdom |  |  |
| Cambridge,England,United+Kingdom |  |  |
| Canterbury,England,United+Kingdom |  |  |
| Carlisle,England,United+Kingdom |  |  |
| Chelmsford,England,United+Kingdom |  |  |
| Chester,England,United+Kingdom |  |  |
| Chichester,England,United+Kingdom |  |  |
| Colchester,England,United+Kingdom |  |  |
| Coventry,England,United+Kingdom |  |  |
| Derby,England,United+Kingdom |  |  |
| Doncaster,England,United+Kingdom |  |  |
| Durham,England,United+Kingdom |  |  |
| Ely,England,United+Kingdom |  |  |
| Exeter,England,United+Kingdom |  |  |
| Gloucester,England,United+Kingdom |  |  |
| Hereford,England,United+Kingdom |  |  |
| Kingston-upon-Hull,England,United+Kingdom |  |  |
| Lancaster,England,United+Kingdom |  |  |
| Leeds,England,United+Kingdom |  |  |
| Leicester,England,United+Kingdom |  |  |
| Lichfield,England,United+Kingdom |  |  |
| Lincoln,England,United+Kingdom |  |  |
| Liverpool,England,United+Kingdom |  |  |
| London,England,United+Kingdom |  |  |
| Manchester,England,United+Kingdom |  |  |
| Milton+Keynes,England,United+Kingdom |  |  |
| Newcastle-upon-Tyne,England,United+Kingdom |  |  |
| Norwich,England,United+Kingdom |  |  |
| Nottingham,England,United+Kingdom |  |  |
| Oxford,England,United+Kingdom |  |  |
| Peterborough,England,United+Kingdom |  |  |
| Plymouth,England,United+Kingdom |  |  |
| Portsmouth,England,United+Kingdom |  |  |
| Preston,England,United+Kingdom |  |  |
| Ripon,England,United+Kingdom |  |  |
| Salford,England,United+Kingdom |  |  |
| Salisbury,England,United+Kingdom |  |  |
| Sheffield,England,United+Kingdom |  |  |
| Southampton,England,United+Kingdom |  |  |
| Southend-on-Sea,England,United+Kingdom |  |  |
| St+Albans,England,United+Kingdom |  |  |
| Stoke+on+Trent,England,United+Kingdom |  |  |
| Sunderland,England,United+Kingdom |  |  |
| Truro,England,United+Kingdom |  |  |
| Wakefield,England,United+Kingdom |  |  |
| Wells,England,United+Kingdom |  |  |
| Winchester,England,United+Kingdom |  |  |
| Wolverhampton,England,United+Kingdom |  |  |
| Worcester,England,United+Kingdom |  |  |
| York,England,United+Kingdom |  |  |
| Armagh,Northern+Ireland,United+Kingdom |  |  |
| Bangor,Northern+Ireland,United+Kingdom |  |  |
| Belfast,Northern+Ireland,United+Kingdom |  |  |
| Lisburn,Northern+Ireland,United+Kingdom |  |  |
| Londonderry,Northern+Ireland,United+Kingdom |  |  |
| Newry,Northern+Ireland,United+Kingdom |  |  |
| Aberdeen,Scotland,United+Kingdom |  |  |
| Dundee,Scotland,United+Kingdom |  |  |
| Dunfermline,Scotland,United+Kingdom |  |  |
| Edinburgh,Scotland,United+Kingdom |  |  |
| Glasgow,Scotland,United+Kingdom |  |  |
| Inverness,Scotland,United+Kingdom |  |  |
| Perth,Scotland,United+Kingdom |  |  |
| Stirling,Scotland,United+Kingdom |  |  |
| Bangor,Wales,United+Kingdom |  |  |
| Cardiff,Wales,United+Kingdom |  |  |
| Newport,Wales,United+Kingdom |  |  |
| St+Asaph,Wales,United+Kingdom |  |  |
| St+Davids,Wales,United+Kingdom |  |  |
| Swansea,Wales,United+Kingdom |  |  |
| Wrexham,Wales,United+Kingdom |  |  |
| Barking+and+Dagenham,London,England,United+Kingdom |  | IG11 |
| Barnet,London,England,United+Kingdom |  | EN4 |
| Bexley,London,England,United+Kingdom |  | DA14 |
| Brent,London,England,United+Kingdom |  | HA0 |
| Bromley,London,England,United+Kingdom |  | BR1 |
| Camden,London,England,United+Kingdom |  | NW1 |
| City+of+London,London,England,United+Kingdom |  |  |
| Croydon,England,United+Kingdom |  | BR3 |
| Ealing,London,England,United+Kingdom |  | W5 |
| Greenwich,London,England,United+Kingdom |  | E14 |
| Hackney,London,England,United+Kingdom |  | E1 |
| Hammersmith+and+Fulham,England,United+Kingdom |  | SW6 |
| Haringey,London,England,United+Kingdom |  | N10 |
| Harrow,London,England,United+Kingdom |  | HA1 |
| Havering,London,England,United+Kingdom |  | RM1 |
| Hillingdon,London,England,United+Kingdom |  | HA2 |
| Hounslow,London,England,United+Kingdom |  | TW4 |
| Islington,London,England,United+Kingdom |  | N1 |
| Royal+Borough+of+Kensington+and+Chelsea,England,United+Kingdom |  | SW10 |
| Kingston+upon+Thames,England,United+Kingdom |  | KT1 |
| Lambeth,London,England,United+Kingdom |  |  |
| Lewisham,London,England,United+Kingdom |  | SE13 |
| Merton,London,England,United+Kingdom |  | CR4 |
| Newham,London,England,United+Kingdom |  | E12 |
| Redbridge,London,England,United+Kingdom |  | E11 |
| Richmond+upon+Thames,England,United+Kingdom |  | KT2 |
| Southwark,London,England,United+Kingdom |  | SE1 |
| Sutton,England,United+Kingdom |  | SM1 |
| Tower+Hamlets,London,England,United+Kingdom |  | E1 |
| Waltham+Forest,London,England,United+Kingdom |  | E10 |
| Wandsworth,London,England,United+Kingdom |  | SW11 |
| Westminster,England,United+Kingdom |  | SW1A |
| Bedfordshire,England,United+Kingdom |  |  |
| Berkshire,England,United+Kingdom |  |  |
| Bristol,England,United+Kingdom |  |  |
| Buckinghamshire,England,United+Kingdom |  |  |
| Cambridgeshire,England,United+Kingdom |  |  |
| Cheshire,England,United+Kingdom |  |  |
| Cornwall,England,United+Kingdom |  |  |
| Cumbria,England,United+Kingdom |  |  |
| Derbyshire,England,United+Kingdom |  |  |
| Devon,England,United+Kingdom |  |  |
| Dorset,England,United+Kingdom |  |  |
| County+Durham,England,United+Kingdom |  |  |
| East+Riding+of+Yorkshire,England,United+Kingdom |  |  |
| East+Sussex,England,United+Kingdom |  |  |
| Essex,England,United+Kingdom |  |  |
| Gloucestershire,England,United+Kingdom |  |  |
| Greater+London,England,United+Kingdom |  |  |
| Greater+Manchester,England,United+Kingdom |  |  |
| Hampshire,England,United+Kingdom |  |  |
| Herefordshire,England,United+Kingdom |  |  |
| Hertfordshire,England,United+Kingdom |  |  |
| Isle+of+Wight,England,United+Kingdom |  |  |
| Kent,England,United+Kingdom |  |  |
| Lancashire,England,United+Kingdom |  |  |
| Leicestershire,England,United+Kingdom |  |  |
| Lincolnshire,England,United+Kingdom |  |  |
| City+of+London,England,United+Kingdom |  |  |
| Merseyside,England,United+Kingdom |  |  |
| Norfolk,England,United+Kingdom |  |  |
| Northamptonshire,England,United+Kingdom |  |  |
| Northumberland,England,United+Kingdom |  |  |
| North+Yorkshire,England,United+Kingdom |  |  |
| Nottinghamshire,England,United+Kingdom |  |  |
| Oxfordshire,England,United+Kingdom |  |  |
| Rutland,England,United+Kingdom |  |  |
| Shropshire,England,United+Kingdom |  |  |
| Somerset,England,United+Kingdom |  |  |
| South+Yorkshire,England,United+Kingdom |  |  |
| Staffordshire,England,United+Kingdom |  |  |
| Suffolk,England,United+Kingdom |  |  |
| Surrey,England,United+Kingdom |  |  |
| Tyne+and+Wear,England,United+Kingdom |  |  |
| Warwickshire,England,United+Kingdom |  |  |
| West+Midlands,England,United+Kingdom |  |  |
| West+Sussex,England,United+Kingdom |  |  |
| West+Yorkshire,England,United+Kingdom |  |  |
| Wiltshire,England,United+Kingdom |  |  |
| Worcestershire,England,United+Kingdom |  |  |
| Antrim,Northern+Ireland,United+Kingdom |  |  |
| Armagh,Northern+Ireland,United+Kingdom | Keady | BT60 |
| Down,Northern+Ireland,United+Kingdom | Downpatrick |  |
| Fermanagh,Northern+Ireland,United+Kingdom | Lisnaskea | BT92 |
| Londonderry,Northern+Ireland,United+Kingdom |  |  |
| Tyrone,Northern+Ireland,United+Kingdom | Omagh |  |
| Angus,Scotland,United+Kingdom | Forfar |  |
| Argyll,Scotland,United+Kingdom | Inveraray |  |
| Ayrshire,Scotland,United+Kingdom | Ayr |  |
| Banffshire,Scotland,United+Kingdom | Banff |  |
| Berwickshire,Scotland,United+Kingdom | Berwick |  |
| Bute,Scotland,United+Kingdom | Rothesay |  |
| Caithness,Scotland,United+Kingdom | Wick |  |
| Clackmannanshire,Scotland,United+Kingdom | Alloa |  |
| Dumfriesshire,Scotland,United+Kingdom | Dumfries |  |
| Dunbartonshire,Scotland,United+Kingdom | Dumbarton |  |
| East+Lothian,Scotland,United+Kingdom | Haddington | EH41 |
| Fife,Scotland,United+Kingdom |  |  |
| Inverness-shire,Scotland,United+Kingdom | Inverness |  |
| Kincardineshire,Scotland,United+Kingdom | Stonehaven | AB39 |
| Kinross-shire,Scotland,United+Kingdom | Kinross |  |
| Kirkcudbrightshire,Scotland,United+Kingdom | Kirkcudbright | DG7 |
| Lanarkshire,Scotland,United+Kingdom | Lanark | ML11 |
| Midlothian,Scotland,United+Kingdom | Dalkeith | EH22 |
| Moray,Scotland,United+Kingdom | Elgin |  |
| Nairnshire,Scotland,United+Kingdom | Nairn |  |
| Orkney,Scotland,United+Kingdom | Kirkwall |  |
| Peeblesshire,Scotland,United+Kingdom | Peebles |  |
| Perthshire,Scotland,United+Kingdom | Perth |  |
| Renfrewshire,Scotland,United+Kingdom | Renfrew |  |
| Ross+and+Cromarty,Scotland,United+Kingdom | Dingwall |  |
| Roxburghshire,Scotland,United+Kingdom | Kelso |  |
| Selkirkshire,Scotland,United+Kingdom | Selkirk | TD7 |
| Shetland,Scotland,United+Kingdom | Lerwick |  |
| Stirlingshire,Scotland,United+Kingdom | Stirling |  |
| Sutherland,Scotland,United+Kingdom | Dornoch |  |
| West+Lothian,Scotland,United+Kingdom |  |  |
| Wigtownshire,Scotland,United+Kingdom | Wigtown | DG8 |
| Blaenau+Gwent,Wales,United+Kingdom |  |  |
| Bridgend,Wales,United+Kingdom |  |  |
| Caerphilly,Wales,United+Kingdom |  |  |
| Camarthenshire,Wales,United+Kingdom | Camarthen | SA31 |
| Ceredigion,Wales,United+Kingdom | Cardigan | SA43 |
| Conwy,Wales,United+Kingdom |  |  |
| Denbighshire,Wales,United+Kingdom | Denbigh |  |
| Flintshire,Wales,United+Kingdom | Flint |  |
| Gwynedd,Wales,United+Kingdom | Bangor |  |
| Isle+of+Anglesey,Wales,United+Kingdom | Holyhead | LL65 |
| Merthyr+Tydfil,Wales,United+Kingdom |  |  |
| Monmouthshire,Wales,United+Kingdom | Monmouth |  |
| Neath+Port+Talbot,Wales,United+Kingdom | Neath |  |
| Pembrokeshire,Wales,United+Kingdom | Haverfordwest |  |
| Powys,Wales,United+Kingdom | Newtown |  |
| Rhondda+Cynon+Taf,Wales,United+Kingdom | Aberdare | CF44 |
| Torfaen,Wales,United+Kingdom | Cwmbran |  |
| Vale+of+Glamorgan,Wales,United+Kingdom | Barry |  |
