# Supplementary material for: Equity, accessibility, and public health implications of digital platforms delivering real-time air quality information: A technology review
Source: PLOS Digit Health. 2026 Apr 17;5(4):e0001280. doi: 10.1371/journal.pdig.0001280 (PMC13089882; doi:10.1371/journal.pdig.0001280)
Supplement: S1 Text — (DOCX) [file pdig.0001280.s004.docx]

# S1 Text. Search terms

Search terms for Google search, Apple IOS, and Google App stores:

- Air quality
- Air quality app
- Air quality forecast
- Air quality nowcast
- Air quality alert
- Air quality map
- Air pollution
- Air pollution app
- Air pollution forecast
- Air pollution nowcast
- Air pollution alert
- Air pollution map
- Smog
- Smog app
- Smog air
- Smog alert
- Smog forecast
- Smog nowcast
- Check air quality
- Air quality today
- Air quality near me
- My AQI air
