# Supplementary figures and images for: Equity, accessibility, and public health implications of digital platforms delivering real-time air quality information: A technology review
Source: PLOS Digit Health. 2026 Apr 17;5(4):e0001280. doi: 10.1371/journal.pdig.0001280 (PMC13089882; doi:10.1371/journal.pdig.0001280)

# S2 Fig. Provider-specific AQ data pipelines


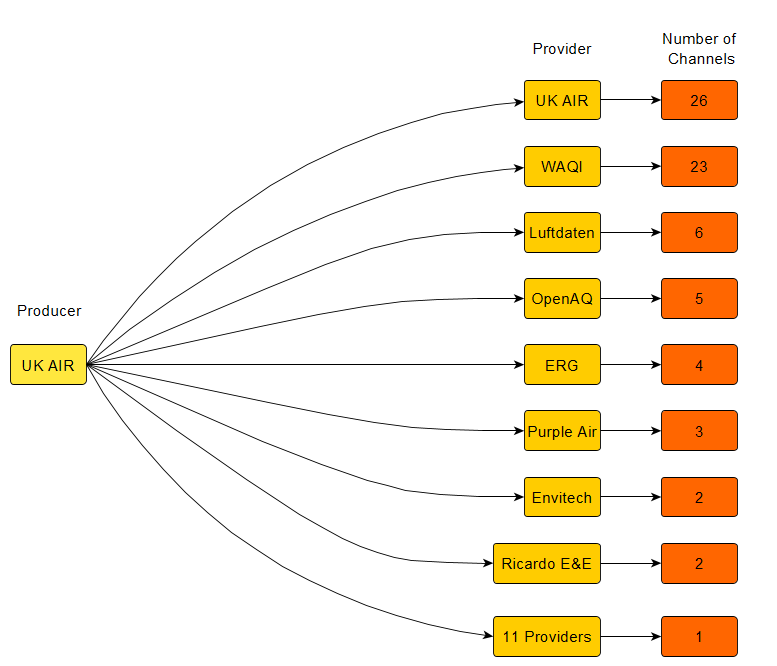


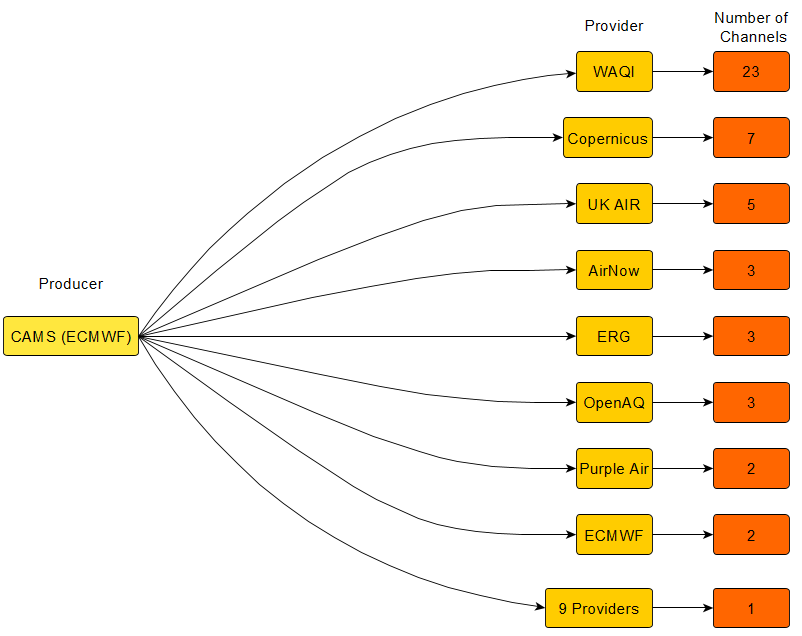


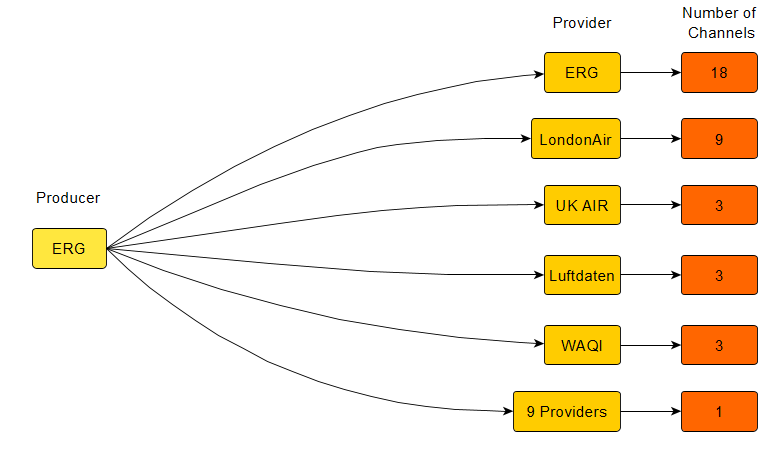


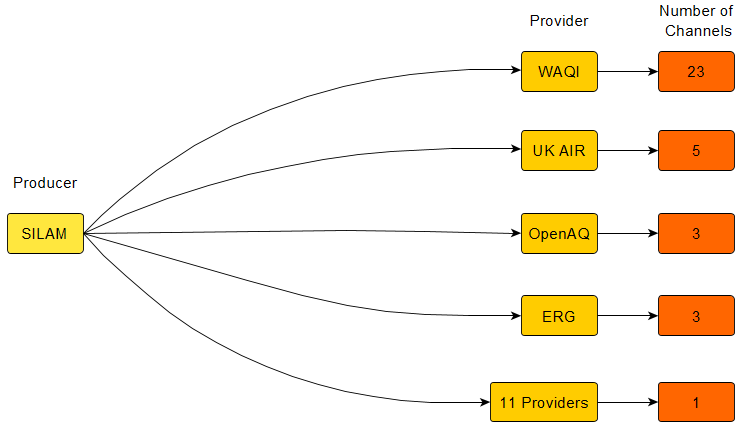


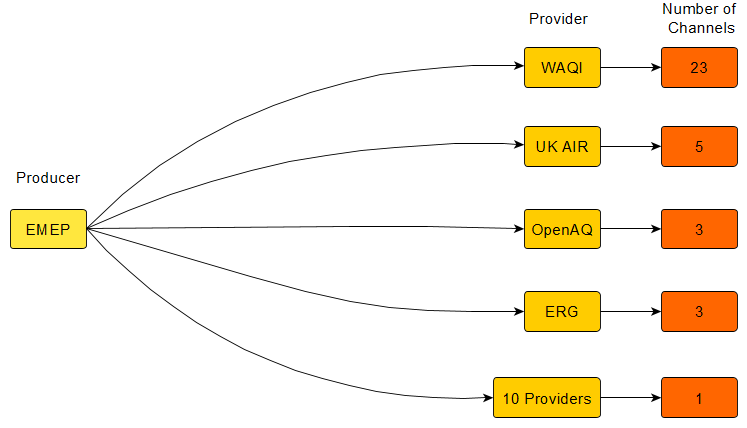


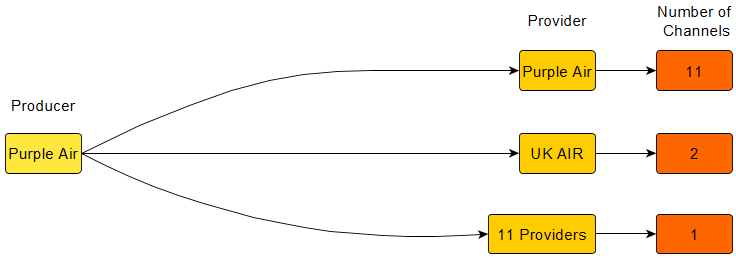

Supplement: S2 Fig — (DOCX) [file pdig.0001280.s005.docx]
