# Supplementary material for: Equity, accessibility, and public health implications of digital platforms delivering real-time air quality information: A technology review
Source: PLOS Digit Health. 2026 Apr 17;5(4):e0001280. doi: 10.1371/journal.pdig.0001280 (PMC13089882; doi:10.1371/journal.pdig.0001280)
Supplement: S2 Table — (DOCX) [file pdig.0001280.s006.docx]

# S2 Table. Tabulated AQ data pipelines

| **Data_Producer** | **Data_Provider** | **Channels** |
| --- | --- | --- |
| Air Quality Egg | Air Quality Egg | Air Quality Egg |
|  | AirBeam | AirCasting \| Air Quality |
| Ambee | Air Quality & Pollen App-Ambee | Air Quality & Pollen App-Ambee |
| AQDM | AQDM | nlincsair.info |
|  | Envitech Ltd | nlincsair.info |
|  | UK Air (Defra) | nlincsair.info |
| Breezometer | Breezometer | Breezometer |
|  | Breezometer | WeatherBug - Radar, Forecast |
| CAMS (ECMWF) | AirNow (EPA) | Air quality app & AQI widget |
|  | AirNow (EPA) | Air quality app & AQI widget |
|  | AirNow (EPA) | wwwen.ipe.org.cn |
|  | Copernicus | Air quality app & AQI widget |
|  | Copernicus | Air quality app & AQI widget |
|  | Copernicus | atmosphere.copernicus.eu |
|  | Copernicus | resourcewatch.org |
|  | Copernicus | Weather - The Weather Channel |
|  | Copernicus | weather.com |
|  | Copernicus | wunderground.com |
|  | ECMWF | Air quality app & AQI widget |
|  | ECMWF | open-meteo.com |
|  | ERG | Air Pollution Monitor - AQI |
|  | ERG | aqicn.org |
|  | ERG | waqi.info |
|  | insdio | waqi.info |
|  | jp2n | waqi.info |
|  | LondonAir | Air Pollution Monitor - AQI |
|  | Luftdaten | AirLief - Air Quality Monitor |
|  | OpenAQ | AirLief - Air Quality Monitor |
|  | OpenAQ | Shoot I Smoke |
|  | OpenAQ | wwwen.ipe.org.cn |
|  | Purple Air | Miasma - Local Air Quality |
|  | Purple Air | wunderground.com |
|  | Smog | AirLief - Air Quality Monitor |
|  | Sofia Air | AirLief - Air Quality Monitor |
|  | SYNGEOS | AirLief - Air Quality Monitor |
|  | The Weather Channel | weather.com |
|  | UK Air (Defra) | Air Pollution Monitor - AQI |
|  | UK Air (Defra) | aqicn.org |
|  | UK Air (Defra) | Miasma - Local Air Quality |
|  | UK Air (Defra) | waqi.info |
|  | UK Air (Defra) | wwwen.ipe.org.cn |
|  | uradmonitors | waqi.info |
|  | WAQI | Air - Pollution around you |
|  | WAQI | Air Pollution Monitor - AQI |
|  | WAQI | Air Quality Global |
|  | WAQI | Air Quality Near Me 2020 |
|  | WAQI | Air Quality Tracker: Pollution |
|  | WAQI | AirLief - Air Quality Monitor |
|  | WAQI | Airveda - Air Quality |
|  | WAQI | aqicn.org |
|  | WAQI | Check Air Quality |
|  | WAQI | Global Air Quality Index-PM2.5 |
|  | WAQI | Haze Today - AQI / API, Pollution & Fire Spots |
|  | WAQI | iAirQuality-global pm2.5,pm11 |
|  | WAQI | Miasma - Local Air Quality |
|  | WAQI | MiseNo - Air Quality Forecast |
|  | WAQI | My AQI Air - PM2.5 / Pollution |
|  | WAQI | Pollen Air Quality |
|  | WAQI | PolluApp by Natur-Air |
|  | WAQI | Pure AQ |
|  | WAQI | Shoot I Smoke |
|  | WAQI | Smog Map Ultra fine particle |
|  | WAQI | waqi.info |
|  | WAQI | Weather Plus: Radar & Forecast |
|  | WAQI | wwwen.ipe.org.cn |
| CERC | CERC | airtext.info |
| Cheltenham (Local Council) | Cheltenham (Local Council) | cheltenham.gov.uk |
| Cornwall (Local Council) | Cornwall (Local Council) | cornwall.gov.uk |
| Coventry (Local Council) | Coventry (Local Council) | coventry.gov.uk |
| Earthsense | BreatheLondon | westminster.gov.uk |
|  | EarthSense | westminster.gov.uk |
|  | ERG | westminster.gov.uk |
| EMEP | AirNow (EPA) | wwwen.ipe.org.cn |
|  | ERG | Air Pollution Monitor - AQI |
|  | ERG | aqicn.org |
|  | ERG | waqi.info |
|  | insdio | waqi.info |
|  | jp2n | waqi.info |
|  | LondonAir | Air Pollution Monitor - AQI |
|  | Luftdaten | AirLief - Air Quality Monitor |
|  | OpenAQ | AirLief - Air Quality Monitor |
|  | OpenAQ | Shoot I Smoke |
|  | OpenAQ | wwwen.ipe.org.cn |
|  | Purple Air | Miasma - Local Air Quality |
|  | Smog | AirLief - Air Quality Monitor |
|  | Sofia Air | AirLief - Air Quality Monitor |
|  | SYNGEOS | AirLief - Air Quality Monitor |
|  | UK Air (Defra) | Air Pollution Monitor - AQI |
|  | UK Air (Defra) | aqicn.org |
|  | UK Air (Defra) | Miasma - Local Air Quality |
|  | UK Air (Defra) | waqi.info |
|  | UK Air (Defra) | wwwen.ipe.org.cn |
|  | uradmonitors | waqi.info |
|  | WAQI | Air - Pollution around you |
|  | WAQI | Air Pollution Monitor - AQI |
|  | WAQI | Air Quality Global |
|  | WAQI | Air Quality Near Me 2020 |
|  | WAQI | Air Quality Tracker: Pollution |
|  | WAQI | AirLief - Air Quality Monitor |
|  | WAQI | Airveda - Air Quality |
|  | WAQI | aqicn.org |
|  | WAQI | Check Air Quality |
|  | WAQI | Global Air Quality Index-PM2.5 |
|  | WAQI | Haze Today - AQI / API, Pollution & Fire Spots |
|  | WAQI | iAirQuality-global pm2.5,pm12 |
|  | WAQI | Miasma - Local Air Quality |
|  | WAQI | MiseNo - Air Quality Forecast |
|  | WAQI | My AQI Air - PM2.5 / Pollution |
|  | WAQI | Pollen Air Quality |
|  | WAQI | PolluApp by Natur-Air |
|  | WAQI | Pure AQ |
|  | WAQI | Shoot I Smoke |
|  | WAQI | Smog Map Ultra fine particle |
|  | WAQI | waqi.info |
|  | WAQI | Weather Plus: Radar & Forecast |
|  | WAQI | wwwen.ipe.org.cn |
| Envitech Ltd | AQDM | nlincsair.info |
|  | Envitech Ltd | airquality.wolverhampton.gov.uk |
|  | Envitech Ltd | cheshireeast.gov.uk |
|  | Envitech Ltd | cheshirewest-air.info |
|  | Envitech Ltd | liverpoolair.org.uk |
|  | Envitech Ltd | nlincsair.info |
|  | Envitech Ltd | nottinghamaqm.net |
|  | Envitech Ltd | southamptonair.org.uk |
|  | Envitech Ltd | ukairquality.net |
|  | Envitech Ltd | worcsregservices.gov.uk |
|  | ERG | hackney.gov.uk |
|  | LondonAir | hackney.gov.uk |
|  | Nottingham AURN | nottinghamaqm.net |
|  | Ricardo E&E | cheshirewest-air.info |
|  | UK Air (Defra) | nlincsair.info |
| ERG | AirAlert | AirAlert |
|  | BreatheLondon | westminster.gov.uk |
|  | City Air | City Air |
|  | EarthSense | westminster.gov.uk |
|  | ERG | Air Pollution Monitor - AQI |
|  | ERG | aqicn.org |
|  | ERG | breathelondon.org |
|  | ERG | brent.gov.uk |
|  | ERG | brighton-hove.gov.uk |
|  | ERG | camden.gov.uk |
|  | ERG | cleanairhub.org.uk |
|  | ERG | ealingair.org.uk |
|  | ERG | hart.gov.uk |
|  | ERG | Lewisham Air |
|  | ERG | lewisham.gov.uk |
|  | ERG | LonData |
|  | ERG | London Air |
|  | ERG | richmond.gov.uk |
|  | ERG | southwark.gov.uk |
|  | ERG | tfl.gov.uk |
|  | ERG | waqi.info |
|  | ERG | westminster.gov.uk |
|  | insdio | waqi.info |
|  | jp2n | waqi.info |
|  | LondonAir | Air Pollution Monitor - AQI |
|  | LondonAir | brent.gov.uk |
|  | LondonAir | camden.gov.uk |
|  | LondonAir | ealingair.org.uk |
|  | LondonAir | hart.gov.uk |
|  | LondonAir | lewisham.gov.uk |
|  | LondonAir | LonData |
|  | LondonAir | richmond.gov.uk |
|  | LondonAir | southwark.gov.uk |
|  | Luftdaten | Air Quality & Pollen - AirCare |
|  | Luftdaten | Breathe - Air Quality Monitor |
|  | Luftdaten | hackAIR |
|  | OpenAQ | hackAIR |
|  | Purple Air | Air Quality & Pollen - AirCare |
|  | UK Air (Defra) | Air Pollution Monitor - AQI |
|  | UK Air (Defra) | aqicn.org |
|  | UK Air (Defra) | waqi.info |
|  | uradmonitors | waqi.info |
|  | WAQI | Air Pollution Monitor - AQI |
|  | WAQI | aqicn.org |
|  | WAQI | waqi.info |
| HabitatMap | Airgradient | openaq.org |
|  | Carnegie Mellon University | openaq.org |
|  | EDF | openaq.org |
|  | Habitat map | openaq.org |
|  | Purple Air | openaq.org |
|  | Senstate | openaq.org |
|  | Smartsense | openaq.org |
| IQAir | IQAir AirVisual \| Air Quality | IQAir AirVisual \| Air Quality |
| Kaiterra | Kaiterra Global | Live Air: Global Air Quality |
| Leicester (Local Council) | Leicester (Local Council) | leicester.gov.uk |
| Luftdaten | Luftdaten | Air Quality & Pollen - AirCare |
|  | Luftdaten | AirLief - Air Quality Monitor |
|  | Luftdaten | Breathe - Air Quality Monitor |
|  | Luftdaten | hackAIR |
|  | Luftdaten | freshairbromley.org.uk |
|  | Luftdaten | sensor.community/en/ |
|  | OpenAQ | AirLief - Air Quality Monitor |
|  | OpenAQ | hackAIR |
|  | Purple Air | Air Quality & Pollen - AirCare |
|  | Smog | AirLief - Air Quality Monitor |
|  | Sofia Air | AirLief - Air Quality Monitor |
|  | SYNGEOS | AirLief - Air Quality Monitor |
|  | WAQI | AirLief - Air Quality Monitor |
| Met Office | Met Office | kentair.org.uk |
|  | Ricardo E&E | kentair.org.uk |
|  | UK Air (Defra) | kentair.org.uk |
|  | InMeteo | ventusky.com |
|  | Met Office | bbc.co.uk |
|  | Met Office | ventusky.com |
|  | Met Office | Weather - Forecasts |
|  | Met Office Weather Forecast | Met Office Weather Forecast |
| N/A | addresspollution.org | addresspollution.org |
|  | Air Pollution Index | Air Pollution Index |
|  | AirChecker, Air quality app | AirChecker, Air quality app |
|  | AirNow (EPA) | The Wildfire Tracker |
|  | AirQApp | AirQApp |
|  | airqualityni.co.uk | airqualityni.co.uk |
|  | AirZen - Real-time Air Quality | AirZen - Real-time Air Quality |
|  | AQI | AQI |
|  | aqi.eco | aqi.eco |
|  | aqli.epic.uchicago.edu | aqli.epic.uchicago.edu |
|  | Digital Health Passport | Digital Health Passport |
|  | Envirotechnology Services | swansea.airqualitydata.com |
|  | Foreca Weather | Foreca Weather |
|  | Local Haze | Local Haze |
|  | Meteum | Meteum |
|  | N/A | AQI Monitor & Weather Forecast |
|  | N/A | Weather - Forecast & Warning |
|  | Smog Alert - Air Pollution | Smog Alert - Air Pollution |
|  | Smog Report | Smog Report |
|  | Smogdog | Smogdog |
|  | Today Weather - Widget & Alert | Today Weather - Widget & Alert |
|  | WEATHER NOW daily forecast app | WEATHER NOW daily forecast app |
|  | ZephAir | ZephAir |
| Nebo | Nebo | Nebo.live |
|  | Purple Air | Nebo.live |
| Newham (Local Council) | Newham (Local Council) | newham.gov.uk |
| Plume Labs | Accuweather | Live Weather Update |
|  | Plume Labs | AccuWeather Weather Forecast |
|  | Plume Labs | Live Weather Update |
|  | Plume Labs | Plume Labs: Air Quality Apps |
| PrevAir | PrevAir | prevair.org |
| Purple Air | AirNow (EPA) | F28AirQuality |
|  | Copernicus | wunderground.com |
|  | Luftdaten | Air Quality & Pollen - AirCare |
|  | Nebo | Nebo.live |
|  | Purple Air | Air Quality & Pollen - AirCare |
|  | Purple Air | Air Quality Reader |
|  | Purple Air | Airly |
|  | Purple Air | Miasma - Local Air Quality |
|  | Purple Air | Nebo.live |
|  | Purple Air | purpleair.com |
|  | Purple Air | Windy.com - Weather & Radar |
|  | Purple Air | wunderground.com |
|  | Purple Air | Air Quality Complication |
|  | Purple Air | F28AirQuality |
|  | UK Air (Defra) | Airly |
|  | UK Air (Defra) | Miasma - Local Air Quality |
|  | WAQI | Miasma - Local Air Quality |
|  | Airgradient | openaq.org |
|  | Carnegie Mellon University | openaq.org |
|  | EDF | openaq.org |
|  | Habitat map | openaq.org |
|  | Purple Air | openaq.org |
|  | Senstate | openaq.org |
|  | Smartsense | openaq.org |
| Ricardo E&E | Air Quality in Scotland | Air Quality in Scotland |
|  | Envitech Ltd | cheshirewest-air.info |
|  | Local Authority | OxonAir |
|  | Met Office | kentair.org.uk |
|  | Met Office | scottishairquality.scot |
|  | NOAA | scottishairquality.scot |
|  | Ricardo E&E | airqualityengland.co.uk |
|  | Ricardo E&E | cheshirewest-air.info |
|  | Ricardo E&E | Edinburgh Air Quality Bot |
|  | Ricardo E&E | kentair.org.uk |
|  | Ricardo E&E | OxonAir |
|  | Ricardo E&E | uBreathe |
|  | Scottish EELG | scottishairquality.scot |
|  | UK Air (Defra) | kentair.org.uk |
|  | UK Air (Defra) | OxonAir |
|  | Windy | scottishairquality.scot |
| Sheffield (Local Council) | Sheffield (Local Council) | sheffield.gov.uk |
| Sheffield University | Sheffield University | sheffieldair.ac.uk |
| SILAM | AirNow (EPA) | wwwen.ipe.org.cn |
|  | ERG | Air Pollution Monitor - AQI |
|  | ERG | aqicn.org |
|  | ERG | waqi.info |
|  | insdio | waqi.info |
|  | jp2n | waqi.info |
|  | LondonAir | Air Pollution Monitor - AQI |
|  | Luftdaten | AirLief - Air Quality Monitor |
|  | OpenAQ | AirLief - Air Quality Monitor |
|  | OpenAQ | Shoot I Smoke |
|  | OpenAQ | wwwen.ipe.org.cn |
|  | OpenWeatherMap | AirQualityMeter |
|  | Purple Air | Miasma - Local Air Quality |
|  | Smog | AirLief - Air Quality Monitor |
|  | Sofia Air | AirLief - Air Quality Monitor |
|  | SYNGEOS | AirLief - Air Quality Monitor |
|  | UK Air (Defra) | Air Pollution Monitor - AQI |
|  | UK Air (Defra) | aqicn.org |
|  | UK Air (Defra) | Miasma - Local Air Quality |
|  | UK Air (Defra) | waqi.info |
|  | UK Air (Defra) | wwwen.ipe.org.cn |
|  | uradmonitors | waqi.info |
|  | WAQI | Air - Pollution around you |
|  | WAQI | Air Pollution Monitor - AQI |
|  | WAQI | Air Quality Global |
|  | WAQI | Air Quality Near Me 2020 |
|  | WAQI | Air Quality Tracker: Pollution |
|  | WAQI | AirLief - Air Quality Monitor |
|  | WAQI | Airveda - Air Quality |
|  | WAQI | aqicn.org |
|  | WAQI | Check Air Quality |
|  | WAQI | Global Air Quality Index-PM2.5 |
|  | WAQI | Haze Today - AQI / API, Pollution & Fire Spots |
|  | WAQI | iAirQuality-global pm2.5,pm13 |
|  | WAQI | Miasma - Local Air Quality |
|  | WAQI | MiseNo - Air Quality Forecast |
|  | WAQI | My AQI Air - PM2.5 / Pollution |
|  | WAQI | Pollen Air Quality |
|  | WAQI | PolluApp by Natur-Air |
|  | WAQI | Pure AQ |
|  | WAQI | Shoot I Smoke |
|  | WAQI | Smog Map Ultra fine particle |
|  | WAQI | waqi.info |
|  | WAQI | Weather Plus: Radar & Forecast |
|  | WAQI | wwwen.ipe.org.cn |
| Sofia Air | Luftdaten | AirLief - Air Quality Monitor |
|  | OpenAQ | AirLief - Air Quality Monitor |
|  | Smog | AirLief - Air Quality Monitor |
|  | Sofia Air | AirLief - Air Quality Monitor |
|  | SYNGEOS | AirLief - Air Quality Monitor |
|  | WAQI | AirLief - Air Quality Monitor |
| Southhampton (Local Council) | Southhampton (Local Council) | southampton.gov.uk |
| SYNGEOS | Luftdaten | AirLief - Air Quality Monitor |
|  | OpenAQ | AirLief - Air Quality Monitor |
|  | Smog | AirLief - Air Quality Monitor |
|  | Sofia Air | AirLief - Air Quality Monitor |
|  | SYNGEOS | AirLief - Air Quality Monitor |
|  | WAQI | AirLief - Air Quality Monitor |
| Tower Hamlets (Local Council) | Tower Hamlets (Local Council) | towerhamlets.gov.uk |
| UK Air (Defra) | AirNow (EPA) | pollution-alert.com |
|  | AirNow (EPA) | wwwen.ipe.org.cn |
|  | AQDM | nlincsair.info |
|  | EEA | EuropeAir |
|  | EEA | weatherandradar.co.uk |
|  | Envitech Ltd | nlincsair.info |
|  | Envitech Ltd | nottinghamaqm.net |
|  | ERG | Air Pollution Monitor - AQI |
|  | ERG | aqicn.org |
|  | ERG | hackney.gov.uk |
|  | ERG | waqi.info |
|  | insdio | waqi.info |
|  | jp2n | waqi.info |
|  | Local Authority | OxonAir |
|  | LondonAir | Air Pollution Monitor - AQI |
|  | LondonAir | hackney.gov.uk |
|  | Luftdaten | Air Quality & Pollen - AirCare |
|  | Luftdaten | AirLief - Air Quality Monitor |
|  | Luftdaten | Breathe - Air Quality Monitor |
|  | Luftdaten | hackAIR |
|  | Luftdaten | freshairbromley.org.uk |
|  | Luftdaten | sensor.community/en/ |
|  | Met Office | kentair.org.uk |
|  | Nottingham AURN | nottinghamaqm.net |
|  | OpenAQ | AirLief - Air Quality Monitor |
|  | OpenAQ | hackAIR |
|  | OpenAQ | pollution-alert.com |
|  | OpenAQ | Shoot I Smoke |
|  | OpenAQ | wwwen.ipe.org.cn |
|  | polleninfo | air-quality.com |
|  | Purple Air | Air Quality & Pollen - AirCare |
|  | Purple Air | Airly |
|  | Purple Air | Miasma - Local Air Quality |
|  | Ricardo E&E | kentair.org.uk |
|  | Ricardo E&E | OxonAir |
|  | Smog | AirLief - Air Quality Monitor |
|  | Sofia Air | AirLief - Air Quality Monitor |
|  | SYNGEOS | AirLief - Air Quality Monitor |
|  | UK Air (Defra) | Air Matters |
|  | UK Air (Defra) | Air Pollution Monitor - AQI |
|  | UK Air (Defra) | Airly |
|  | UK Air (Defra) | air-quality.com |
|  | UK Air (Defra) | AQ+ AirQuality & Weather Chat |
|  | UK Air (Defra) | aqicn.org |
|  | UK Air (Defra) | Clean Air in Cities |
|  | UK Air (Defra) | derbyshiredales.gov.uk |
|  | UK Air (Defra) | Dusty - Particulate Matter |
|  | UK Air (Defra) | exeter.gov.uk |
|  | UK Air (Defra) | Haze: Air Quality & Weather |
|  | UK Air (Defra) | heathrowairwatch.org.uk |
|  | UK Air (Defra) | hillingdon-air.info |
|  | UK Air (Defra) | kentair.org.uk |
|  | UK Air (Defra) | leeds.gov.uk |
|  | UK Air (Defra) | llyw.cymru |
|  | UK Air (Defra) | Miasma - Local Air Quality |
|  | UK Air (Defra) | nlincsair.info |
|  | UK Air (Defra) | OxonAir |
|  | UK Air (Defra) | pollution-alert.com |
|  | UK Air (Defra) | smogwatch.co.uk |
|  | UK Air (Defra) | sussex-air.net |
|  | UK Air (Defra) | uk-air.defra.gov.uk |
|  | UK Air (Defra) | waqi.info |
|  | UK Air (Defra) | wecare4air.co.uk |
|  | UK Air (Defra) | wwwen.ipe.org.cn |
|  | uradmonitors | waqi.info |
|  | WAQI | Air - Pollution around you |
|  | WAQI | Air Pollution Monitor - AQI |
|  | WAQI | Air Quality Global |
|  | WAQI | Air Quality Near Me 2020 |
|  | WAQI | Air Quality Tracker: Pollution |
|  | WAQI | AirLief - Air Quality Monitor |
|  | WAQI | Airveda - Air Quality |
|  | WAQI | aqicn.org |
|  | WAQI | Check Air Quality |
|  | WAQI | Global Air Quality Index-PM2.5 |
|  | WAQI | Haze Today - AQI / API, Pollution & Fire Spots |
|  | WAQI | iAirQuality-global pm2.5,pm10 |
|  | WAQI | Miasma - Local Air Quality |
|  | WAQI | MiseNo - Air Quality Forecast |
|  | WAQI | My AQI Air - PM2.5 / Pollution |
|  | WAQI | Pollen Air Quality |
|  | WAQI | PolluApp by Natur-Air |
|  | WAQI | Pure AQ |
|  | WAQI | Shoot I Smoke |
|  | WAQI | Smog Map Ultra fine particle |
|  | WAQI | waqi.info |
|  | WAQI | Weather Plus: Radar & Forecast |
|  | WAQI | wwwen.ipe.org.cn |
| University of Basel | University of Basel | meteoblue.com |
