# Supplementary material for: Equity, accessibility, and public health implications of digital platforms delivering real-time air quality information: A technology review
Source: PLOS Digit Health. 2026 Apr 17;5(4):e0001280. doi: 10.1371/journal.pdig.0001280 (PMC13089882; doi:10.1371/journal.pdig.0001280)
Supplement: S2 Text — (DOCX) [file pdig.0001280.s007.docx]

# S2 Text. Metadata list

Metadata was gathered for the following fields (where available):

- Channel name
- Channel provider
- URL
- Channel launch date
- Delivery mechanism (website, mobile app, radio, television)
- What hardware is it available on (computer, phone, tablet, television, smartwatch)
- If mobile app, which provider (Android, iOS, or both)
- Does the channel offer an API?
- Can it be paired with a personal sensor?
- AQ data source (model, monitor or sensor)
- If model, what type of model (Proprietary or existing government model)
- Model name (if available)
- Geographic resolution of the estimate or measurement
- Temporal frequency of measurements
- Temporal frequency of model updates
- AQ data provider (if different from channel provider)
- Is a forecast available?
- How many days ahead forecasted?
- Is historic data available?
- What pollutants are reported
- Is an AQI shown?
- If an AQI is shown, which one(s)?
- Latest update
- Returned data or reading in the past year?
- Volume of access
- Total downloads (via Google store)
- Total visits in past year (Google analytics/social media views and followers)
- Region (UK or globally available?)
- Are exposure reducing behavioural (ERB) messaging or recommendations provided?
- What messages are provided?
- Are sensitive groups identified?
- How are sensitive groups defined
- Are push notifications available?
- Paid option?

App store metadata

Table A below shows which metadata information was provided across Google Play and Apple iOS app stores.

*Table A. App store metadata*

| App Store | Title | Link | Product ID | Description | Category | Release Date | Price | Downloads |
| --- | --- | --- | --- | --- | --- | --- | --- | --- |
| Apple |  |  |  |  |  |  |  |  |
| Google Play |  |  |  |  |  |  |  |  |
